# The generic Markov cohomological Hall algebra is not spherically generated

**DOI:** 10.1098/rsos.250282

**Published:** 2025-08-13

**Authors:** Ben Davison

**Affiliations:** ^1^ School of Mathematics and Maxwell Institute for Mathematical Sciences, Edinburgh University, Edinburgh, UK

**Keywords:** cohomological Hall algebras, cluster algebras, BPS invariants

## Abstract

Let Q be the Markov quiver, and let W be an infinitely mutable potential for Q. We calculate some low-degree refined Bogomol'nyi-Prasad-Sommerfield (BPS) invariants for the resulting Jacobi algebra and use them to show that the critical cohomological Hall algebra $H_{Q,W}$ is not necessarily spherically generated and is not independent of the choice of infinitely mutable potential W. This leads to a counterexample to a conjecture of Gaiotto *et al*. (Gaiotto *et al*. 2024 Categories of line defects and cohomological Hall algebras. *arXiv*. §2.1), but also suggestions for how to modify it. In the case of generic cubic W, we discuss a way to modify the conjecture by excluding the non-spherical part via the decomposition of $H_{Q,W}$ according to the characters of a discrete symmetry group.

## 1. Preliminaries

Given a quiver Q with potential W∈ℂQ/[ℂQ,ℂQ], the Kontsevich–Soibelman cohomological Hall algebra (CoHA) $H_{Q,W}$ is an associative algebra that provides a beautiful link between two worlds (see [1] for details). On the one hand, taking partition functions encoding the dimensions of the graded pieces of $H_{Q,W}$ and factorizing them according to the slopes determined by a given stability condition, we may extract the *refined BPS invariants* of the category of representations for the Jacobi algebra Jac(Q,W) associated to Q and W. These invariants have their origins in physics and should be thought of as counting BPS states on the noncommutative Calabi–Yau threefold associated to Jac(Q,W).

On the other hand, $H_{Q,W}$ is an *algebra*, and for suitable choices of Q and W, this algebra can be shown to recover and extend various quantum groups and may be used to prove new results regarding Yangian-type algebras [2].

On the algebraic side, the quivers Q with potential for which the algebra $H_{Q,W}$ has been most intensively studied are *symmetric*, meaning that for every pair of vertices i and j in Q, there are as many arrows from i to j as there are from j to i. From the point of view of studying BPS invariants, this is quite a restrictive set of quivers: it is the set of quivers for which the BPS invariants are independent of stability conditions, and all wall-crossing phenomena disappear. Also, from the point of view of cluster algebras, the class of symmetric quivers is an unnatural choice, since in that subject (see [3] for background) the usual restriction on quivers is that they contain no loops or two-cycles. A symmetric quiver satisfying these restrictions has no arrows at all!

This short paper is inspired by a pair of related conjectures in [4, §2.1]. The first states that if W is an infinitely mutable^1^ potential for a quiver Q containing no loops or two-cycles, then $H_{Q,W}≅S_{Q}$, where $S_{Q}$ is the spherical subalgebra of the shuffle algebra $H_{Q}$ (see §3 for partial definitions). Note that, while calculations inside $H_{Q,W}$ are made rather difficult by the necessity of working with vanishing cycle cohomology, the algebra $H_{Q}$ has a very down-to-earth presentation, and it may be studied and understood, along with its subalgebra $S_{Q}$, using elementary calculations and computer algebra packages. So it would be excellent news to discover that the algebra $H_{Q,W}$, for which it is hard to calculate products and for which the graded dimensions recover refined BPS invariants, is in fact isomorphic to $S_{Q}$. The weaker version of this conjecture, also stated in [4, §2.1], states that $H_{Q,W}$ is independent of W, as long as W is chosen to be infinitely mutable.

The *Markov quiver*, for which the definition is recalled in §2, has a well-established reputation as a source of interesting properties, examples and counterexamples in the theory of cluster algebras (see [3,5] and references therein). The study of the cluster algebra built from this quiver is closely connected to the study of solutions to Markov’s equation; we refer to [6] for recent work in this direction, along with further references. True to its reputation, in this short paper, we present counterexamples to the above conjectures (propositions 3.2 and 3.4), built from the Markov quiver with infinitely mutable potentials. More positively, we will see that the Markov quiver provides example calculations that suggest how the spherical generation conjecture might be modified.

### 1.1. Set-up

By a quiver Q we mean a finite directed graph. We set $Q_{0}$ to be the set of vertices of Q, $Q_{1}$ to be the set of arrows and $s,t:Q_{1}\to Q_{0}$ to be the two morphisms sending an arrow to its source and target, respectively. Let $d\in {\mathbb{N}}^{Q_{0}}$ be a dimension vector. We denote by $M_{d}(Q)$ the stack of d-dimensional ℂQ-modules. It has dimension $-\chi_{Q}(d,d)$*,* where $\chi_{Q}$ is the *Euler form* defined by


$$\begin{array}{rl} \chi_{Q}: & N^{Q_{0}}\times N^{Q_{0}}\to Z\\ & (d,e)\mapsto \sum_{i\in Q_{0}}d_{i}e_{i}-\sum_{a\in Q_{1}}d_{t(a)}e_{s(a)}. \end{array}$$

We can present the stack $M_{d}(Q)$ as a global quotient stack, as we briefly recall. We define $A_{d}(Q):=\prod_{a\in Q_{1}}\text{Hom}(C^{d_{s(a)}},C^{d_{t(a)}})$, a vector space parameterizing d-dimensional ℂQ-modules, which we may consider as an affine variety in the obvious way. This is acted on by the gauge group ${GL}_{d}:=\prod_{i\in Q_{0}}{\text{GL}}_{d_{i}}(C)$ by a simultaneous change of basis. Then $M_{d}(Q)≅A_{d}(Q)/{GL}_{d}$.

Let W∈ℂQ/[ℂQ,ℂQ] be a linear combination of (finitely many) cyclic paths. Taking the trace of W, considered as an endomorphism of the underlying vector spaces of ℂQ-modules, provides a function Tr(W) on $M_{d}(Q)$. It is often desirable, in the study of mutation of quivers with potentials, to consider formal potentials (infinite linear combinations of cyclic paths). In order to define the CoHA, we will not do so, so that the function Tr(W) is well defined.

The Kontsevich–Soibelman critical CoHA $H_{Q,W}$ is a ${\mathbb{N}}^{Q_{0}}$-graded associative algebra, for which the underlying vector space of the dth graded piece is the vanishing cycle cohomology


$$H_{Q,W,d}=H(M_{d}(Q),\phi_{Tr(W)}Q[-\chi_{Q}(d,d)]),$$

and the square brackets denote the cohomological shift. In words, the dth graded piece of $H_{Q,W}$ is the hypercohomology of the perverse sheaf of vanishing cycles for the function Tr(W) on the stack of d-dimensional Q-representations.

The associative product $m:H_{Q,W}^{⊗2}\to H_{Q,W}$ is defined in [1, §7]. We remark that the product respects the cohomological grading on $H_{Q,W}$ if and only if Q is symmetric. In general, the failure of the CoHA multiplication to preserve the cohomological grading is captured by the following formula relating cohomological degrees, where we assume $\alpha \in H_{Q,W,d}$ and $\beta \in H_{Q,W,e}$, and we use ∘ to denote the CoHA multiplication


(1.1) $$|\alpha \circ \beta |=|\alpha |+|\beta |+\chi_{Q}(d,e)-\chi_{Q}(e,d).$$

Fix a quiver Q. We define the ring $A_{Q}$ as follows. It is a $\mathbb{Z}(\;(q^{1/2})\;)$-module, and as a $\mathbb{Z}(\;(q^{1/2})\;)$-module, it is equal to the set of formal linear combinations $\sum_{d\in N^{Q_{0}}}a_{d}(q^{1/2})x^{d}$ with each $a_{d}(q^{1/2})\in \mathbb{Z}(\;(q^{1/2})\;)$. The multiplication is given by extending the rule $x^{d}x^{e}=(-q^{1/2}{)}^{\chi_{Q}(d,e)-\chi_{Q}(e,d)}x^{d+e}$ to formal linear combinations.

We consider the partition function in $A_{Q}$


$$Z_{Q,W}(x):=\sum_{d\in N^{Q_{0}}}\chi_{q^{1/2}}\left(H_{Q,W,d}\right)x^{d},$$

where for a ℤ-graded vector space V we set


$$\chi_{q^{1/2}}(V):=\sum_{n\in Z}\dim(V^{n})(-q^{1/2}{)}^{n}.$$

**Remark 1.1.** *Conceptually, it often makes more sense to replace the above Poincaré series with a ‘weight’ Poincaré series that is sensitive to the mixed Hodge structure on* $H_{Q,W}$ *and, in particular, the weight filtration. See* [1, §7] *for definitions and details of this approach. Since in this paper we will only be interested in calculating graded dimensions of certain vector spaces, we ignore this alternative and instead take naive Poincaré series throughout.*

Let $\zeta \in Q^{Q_{0}}$ be a *stability condition*. We define the *slope* μ(d) of a dimension vector $d\in {\mathbb{N}}^{Q_{0}}\setminus \left\{0\right\}$ by setting


$$\mu (d)=\frac{d\cdot \zeta }{\sum_{i\in Q_{0}}d_{i}}.$$

We assume that ζ is *generic*, meaning that if $d,e\in {\mathbb{N}}^{Q_{0}}\setminus \left\{0\right\}$ have the same slope, then $\chi_{Q}(d,e)=\chi_{Q}(e,d)$. Given a slope θ∈(−∞,∞), we define


$$\Lambda_{\theta }^{\zeta }:=\left\{d\in N^{Q_{0}}|d=0\text{ or }\mu (d)=\theta \right\}.$$

We define $A_{Q,\theta }$ to be the subring of $A_{Q}$ spanned by formal $\mathbb{Z}(\;(q^{1/2})\;)$-linear combinations of symbols $x^{d}$, where $d\in \Lambda_{\theta }^{\zeta }$. By the genericity of ζ, for every θ, the ring $A_{Q,\theta }$ is commutative. There is a unique factorization


(1.2) $$Z_{Q,W}(x)=\prod_{\infty \overset{\theta }{\to }-\infty }Z_{Q,W,\theta }^{\zeta }(x),$$

where $Z_{Q,W,\theta }^{\zeta }(x)\in A_{Q,\theta }$. By the cohomological wall-crossing isomorphism [7, Thm. B], there are equalities


(1.3) $$Z_{Q,W,\theta }^{\zeta }(x)=\sum_{d\in \Lambda_{\Theta }^{\zeta }}\chi_{q^{1/2}}\left(H\left(M_{d}^{\zeta -ss}(Q),\phi_{\mathrm{Tr}(W)}Q\left[-\chi_{Q}(d,d)\right]\right)\right)x^{d},$$

where $M_{d}^{\zeta -ss}(Q)\subset M_{d}(Q)$ is the substack of ζ-semistable Q-representations.

We may repackage the functions $Z_{Q,W,\theta }^{\zeta }(x)$ in terms of *refined BPS invariants*, which are Laurent polynomials $\Omega_{Q,W,d}^{\zeta }\in Z[q^{\pm 1/2}]$ defined via the equality


(1.4) $$Z_{Q,W,\theta }^{\zeta }(x)=\mathrm{Exp}\left(\sum_{d\in \Lambda_{\theta }^{\zeta }\setminus \{0\}}\Omega_{Q,W,d}^{\zeta }x^{d}\left(-q^{1/2}\right)(1-q{)}^{-1}\right).$$

Here Exp is the plethystic exponential, defined by setting


$$\begin{array}{r} \mathrm{Exp}\left(\sum_{\begin{array}{c} d\in \Lambda_{\hat{\Theta }}^{\zeta }\setminus \{0\}\\ n\in Z \end{array}}a_{d,n}x^{d}q^{n/2}\right):=\prod_{\begin{array}{c} d\in \Lambda_{\hat{\Theta }}^{\zeta }\setminus \{0\}\\ n\in Z \end{array}}{\left(1-q^{n/2}x^{d}\right)}^{-a_{d,n}} \end{array}$$

whenever the right-hand side makes sense. The fact that the formal power series $\Omega_{Q,W,d}^{\zeta }\in Z(\;(q^{1/2})\;)$ defined by (1.4) are actually Laurent polynomials is a consequence of the cohomological integrality theorem [7, Thm. A].

**Remark 1.2*.*** *The polynomials* $\Omega_{Q,W,d}^{\zeta }$ *can be realized by taking the Poincaré polynomials of BPS cohomology, introduced in* [7]. *If we had defined the partition functions* $Z_{Q,W,\theta }^{\zeta }(x)$ *using weight series instead, we would take the weight polynomials of BPS cohomology to recover the corresponding refined BPS invariants. Since in this paper we are principally interested in the dimensions of vector spaces, it is most natural to consider naive Poincaré series.*

### 1.2. The conjectures

Fixing a quiver Q, it is very interesting to study the dependence of $Z_{Q,W}(x)$ on W. It is conjectured in [4, §2.1] that as long as the quiver with potential (Q,W) is *infinitely mutable*, the partition function $Z_{Q,W}(x)$ does not depend on the choice of W. This is equivalent to the statement that after fixing a stability condition ζ, the BPS invariants $\Omega_{Q,W,d}^{\zeta }$ do not depend on W. Being infinitely mutable is a certain non-degeneracy condition on quivers with potentials that is important in the categorification of cluster algebras via Ginzburg’s differential graded algebras (see [3,8,9] for definitions, motivation and background). It is, first of all, assumed that Q does not contain loops and two-cycles. Then the mutation at a given vertex i produces a new quiver with potential^2^ $\mu_{i}(Q,W)$. Infinite mutability is the condition that the underlying quiver of this mutated quiver with potential also does not contain two cycles, and that this remains the case after iterated mutation at any sequence of vertices.

## 2. The Markov quiver

### 2.1. Potentials for the Markov quiver

For the rest of the paper, we fix Q to be the Markov quiver. Precisely, we set $Q_{0}=\left\{1,2,3\right\}$ and $Q_{1}=\left\{a_{1},a_{2},b_{1},b_{2},c_{1},c_{2}\right\}$*,* with the orientations of the arrows as in the following diagram:


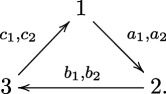


Let W∈ℂQ/[ℂQ,ℂQ] be a potential. We grade ℂQ by path length.

**Lemma 2.1.** [10, chapter 14, example 4.5] *After applying a graded linear isomorphism* Φ:ℂQ→ℂQ*, i.e. an isomorphism taking arrows to linear combinations of arrows, we may write* W *in one of the following five forms:*

(1) $W=W_{\geq 6}$
(2) $W=c_{1}b_{1}a_{1}+W_{\geq 6}$
(3) $W=c_{1}b_{1}a_{1}+c_{1}b_{2}a_{2}+W_{\geq 6}$
(4) $W=c_{1}b_{1}a_{2}+c_{1}b_{2}a_{1}+c_{2}b_{1}a_{1}+W_{\geq 6}$
(5) $W=c_{1}b_{1}a_{1}+c_{2}b_{2}a_{2}+W_{\geq 6}$*,*

*where* $W_{\geq 6}$ *is the sum of all of the homogeneous pieces of* W *of degree at least* 6*, i.e. a linear combination of cyclic paths of length at least* 6.

*Moreover, case (5)* *is generic, in the following sense: the type of a potential* W *under graded linear isomorphisms is determined by the cubic part of* W*, and a generic homogeneous cubic potential* W *can be transformed to the form* $W=c_{1}b_{1}a_{1}+c_{2}b_{2}a_{2}$.

**Proposition 2.2.** W *is infinitely mutable if and only if it is of one of the forms given in cases (4) and (5) above.*

*Proof*. In the first three cases, a single mutation at vertex 2 produces a quiver with potential that contains at least one two-cycle (see [3] for the definition of mutation for quivers with potentials). So we just need to show that in the remaining two cases, the quiver with potential is infinitely mutable. For case (5), this is [3, example 8.6]. The argument for case (4) is the same as the argument for (5); we write it for completeness.

As in [3], we consider W as an element in the *completed* path algebra $\prod_{n\geq 0}(\mathbb{C}Q{)}_{n}$, where $(\mathbb{C}Q{)}_{n}$ is the vector space spanned by paths of length n. We describe the mutation of (Q,W) at vertex 2 and show that the mutated quiver has no two-cycles; this will suffice, since the quiver with potential is invariant under rotational symmetry by the group ℤ/3ℤ. The new quiver has arrows $a_{1}^{*},a_{2}^{*}$ from 2 to 1, arrows $b_{1}^{*},b_{2}^{*}$ from 3 to 2, arrows $c_{1},c_{2}$ from 3 to 1 and four arrows $\left[b_{i}a_{j}\right]$ from 1 to 3, with i,j∈{1,2}. The new potential, before cancelling off quadratic terms, is of the form


$$W^{\prime }=c_{1}[b_{1}a_{2}]+c_{1}[b_{2}a_{1}]+c_{2}[b_{1}a_{1}]+\sum_{i,j\in \{1,2\}}[b_{i}a_{j}]a_{j}^{*}b_{i}^{*}+W_{\geq 4}^{\prime }.$$

Writing $u=[b_{1}a_{2}]+\left[b_{2}a_{1}\right]$ and $v=[b_{1}a_{2}]-\left[b_{2}a_{1}\right]$ we find


$$W^{\prime }=c_{1}u+c_{2}[b_{1}a_{1}]+[b_{1}a_{1}]a_{1}^{*}b_{1}^{*}+[b_{2}a_{2}]a_{2}^{*}b_{2}^{*}+(u+v)a_{2}^{*}b_{1}^{*}/2+(u-v)a_{1}^{*}b_{2}^{*}/2+W_{\geq 4}^{\prime }.$$

As in [3, definition 2.5], we define a *unitriangular* automorphism ψ of the (completed) path algebra to be one for which, for every arrow $a\in Q_{1}$, $\psi (a)=a+\psi (a{)}_{\geq 2}$, where $\psi (a{)}_{\geq 2}$ is a formal linear combination of paths of length at least two. By [3, lemma 4.7], there is a unitriangular automorphism sending $c_{1}\mapsto c_{1}-a_{2}^{*}b_{1}^{*}/2-a_{1}^{*}b_{2}^{*}/2+\ldots$ and $c_{2}\mapsto c_{2}-a_{1}^{*}b_{1}^{*}+\ldots$, such that after rescaling v and $a_{1}^{*}$, the potential $W^{\prime }$ transforms to


$$W^{\prime \prime }=c_{1}u+c_{2}[b_{1}a_{1}]+[b_{2}a_{2}]a_{2}^{*}b_{2}^{*}+va_{2}^{*}b_{1}^{*}+va_{1}^{*}b_{2}^{*}+W_{\geq 6}^{\prime \prime },$$

where $W_{\geq 6}^{\prime \prime }$ does not contain the arrows $c_{1},u,c_{2},\left[b_{1}a_{1}\right]$. Removing the quadratic terms and the two-cycles $c_{1}u$ and $c_{2}[b_{1}a_{1}]$, we find that the mutated quiver with potential contains no two-cycles, is isomorphic to the Markov quiver and the new potential is again of the form (4).∎

### 2.2. BPS invariants for generic W and small dimension vectors

In order to use the formulas (1.2) and (1.3), we need to make a choice of stability condition. For the rest of the paper, we fix a stability condition $\zeta \in Q^{Q_{0}}$ by setting $\zeta_{1}=1$, $0<\zeta_{2}=\epsilon \ll 1$ with ϵ irrational and $\zeta_{3}=-1$, although the calculation of the refined BPS invariants for small dimensions and other choices of generic stability conditions is performed in the same way.

Next, we calculate some low-degree refined BPS invariants for potentials of generic form ((5) above). Setting $d=d\delta_{i}$ to be the dimension vector that is zero everywhere apart from $i\in Q_{0}$ and for which the entry at i is d, we find $M_{d}^{\zeta -ss}(Q)≅pt/{GL}_{d}(C)$ and the function Tr(W) is zero on this stack. So if θ=1,ϵ,−1*,* we have the standard calculation


$$\begin{array}{rl} Z_{Q,W,\theta }^{\zeta }(x)= & \sum_{d\geq 0}\chi_{q^{1/2}}(H(\text{pt}/{\text{GL}}_{d}(C),Q[-d^{2}]))x^{d\delta_{i}}\\ = & \text{Exp}\left(x^{\delta_{i}}\frac{-q^{1/2}}{\left(1-q\right)}\right). \end{array}$$

In particular,


$$\Omega_{Q,W,\delta_{i}}^{\zeta }=1$$

for i=1,2,3.

Now let d=(1,1,0). A ζ-stable d-dimensional Q-representation is given by two linear maps $\rho (a_{1}):\mathbb{C}\to \mathbb{C}$ and $\rho (a_{2}):\mathbb{C}\to \mathbb{C}$, satisfying the condition that neither of them is the zero map. We thus see that $M_{d}^{\zeta -ss}(Q)≅P^{1}/C^{*}$. Again, the function Tr(W) is zero on this stack, and we have the isomorphism of sheaves $\phi_{\text{Tr}(W)}Q_{P^{1}/C^{*}}[-\chi_{Q}(d,d)]≅Q_{P^{1}/C^{*}}$. Comparing (1.3) and (1.4), we deduce


$$\Omega_{Q,W,(1,1,0)}^{\zeta }=-q^{-1/2}-q^{1/2},$$

which is the normalized Poincaré polynomial of ${\mathbb{P}}^{1}$. By the same argument, we calculate


$$\Omega_{Q,W,(0,1,1)}^{\zeta }=-q^{-1/2}-q^{1/2}.$$

On the other hand, there are no ζ-semistable Q-representations of dimension vector (1,0,1); such a module ρ would have a destabilizing submodule of dimension vector (1,0,0). So it follows, again from (1.3), that


$$\Omega_{Q,W,(1,0,1)}^{\zeta }=0.$$

Note that, so far, our choice of W has played essentially no role. More generally, it follows from the discussion in [11, §8.4] that, for infinitely mutable potentials for the Markov quiver Q, the BPS invariants $\Omega_{Q,W,d}^{\zeta }$ for d∉ℕ⋅(1,1,1) are entirely determined by the rules governing the behaviour of $Z_{Q,W}(x)$ under cluster mutation and do not depend on the potential W. Our choice of potential can only start to be relevant for the dimension vector (1,1,1).

**Proposition 2.3.** *Continue to assume that* W *is generic, i.e. that we can write* $W=c_{1}b_{1}a_{1}+c_{2}b_{2}a_{2}+W_{\geq 6}$. *Then*

— $\Omega_{Q,W,(1,1,1)}^{\zeta }=2+e(q^{1/2})$*, where* $e(q^{1/2})\in \mathbb{N}[(-q^{1/2}{)}^{\pm 1}]$ *is a Laurent polynomial in* $q^{1/2}$*, with the coefficient of* $q^{n/2}$ *positive or negative depending on whether* n *is even or odd.*
— *If we set* $W=c_{1}b_{1}a_{1}+c_{2}b_{2}a_{2}$, *then* $\Omega_{Q,W,(1,1,1)}^{\zeta }=2$*.*

*Proof*. Let ρ be a ζ-semistable (1,1,1)-dimensional Q-representation. Fixing identifications between the vector spaces that ρ assigns to the three vertices and the one-dimensional vector space ℂ, ρ is determined by six linear maps $\rho (a_{1}),\rho (a_{2}),\ldots$, which we may identify with numbers in ℂ. We abuse notation by denoting these numbers $a_{1},\ldots ,c_{2}$. Then stability for ρ is equivalent to the two conditions

— At least one of $a_{1},a_{2}$ is non-zero.
— At least one of $b_{1},b_{2}$ is non-zero.

Let $M=M_{\left(1,1,1\right)}^{\zeta -ss}(Q)$ be the coarse moduli space; since (1,1,1) is indivisible, this is a fine moduli space, and moreover, we have $M_{\left(1,1,1\right)}^{\zeta -ss}(Q)≅M/C^{*}$, the quotient by the trivial ${\mathbb{C}}^{*}$-action.

We cover M by the four charts $M_{i,j}$, for i,j∈{1,2}, where $M_{i,j}$ is defined to be the subvariety corresponding to Q-representations for which $a_{i}\neq 0$ and $b_{j}\neq 0$. Then each of $M_{i,j}$ is isomorphic to $A^{4}$; up to gauge equivalence $a_{i}=1,b_{j}=1$, and then the remaining four arrows provide the four coordinates of affine four-space. We prove the final part of the proposition first, so for now we set $W=c_{1}b_{1}a_{1}+c_{2}b_{2}a_{2}$. I claim that


$$\phi_{\text{Tr}(W)}Q_{M}[4]{|}_{M_{i,j}}=\left\{ \begin{array}{ll} 0 & \text{if }i=j\\ Q_{0} & \text{if }i\neq j. \end{array}\right.$$

The first case (i=j) is easy: in local coordinates, we write


$$\text{Tr}(W)=c_{i}+c_{k}b_{k}a_{k},$$

with k≠i. In particular, the critical locus of this function is empty, and since $\phi_{\text{Tr}(W)}Q_{M_{i,j}}[4]$ is supported on this locus, the first part of the claim follows.

For the second case (i≠j), we have instead


(2.1) $$\text{Tr}(W)=c_{j}a_{i}+c_{i}b_{j}.$$

By the Thom–Sebastiani isomorphism [12], we find


$$\phi_{\text{Tr}(W)}Q_{M_{i,j}}[4]≅\phi_{c_{j}a_{i}}Q_{A^{2}}[2]⊠\phi_{c_{i}a_{j}}Q_{A^{2}}[2]≅Q_{0}⊠Q_{0}.$$

We have used here the standard calculation $\phi_{xy}Q_{A^{2}}[2]≅Q_{0}$, where ${\mathbb{Q}}_{0}$ is the constant sheaf supported on the origin $0\in A^{2}$.

Let α∈M be the point corresponding to the module for which $a_{1}$ and $b_{2}$ act via isomorphisms and all other arrows act via the zero map. Let β∈M be the point corresponding to the module for which $a_{2}$ and $b_{1}$ act via isomorphisms and all other arrows act via the zero map. We depict them as follows:


(2.2) $$\begin{array}{ll} \alpha : & 1\overset{a_{1}}{\to }2\overset{b_{2}}{\to }3\\ \beta : & 1\overset{a_{2}}{\to }2\overset{b_{1}}{\to }3. \end{array}$$

The claim tells us that $\phi_{\text{Tr}(W)}Q_{M}[4]≅Q_{\alpha }⊕Q_{\beta }$.

Then we have


$$\begin{array}{rl} H(M_{\left(1,1,1\right)}^{\zeta -ss}(Q),\phi_{\text{Tr}(W)}Q[3])≅ & H(M_{\left(1,1,1\right)}^{\zeta -ss}(Q),\phi_{\text{Tr}(W)}Q[4])⊗H(pt/C^{*},Q[-1])\\ ≅ & H(\alpha \cup \beta ,Q)⊗H(pt/C^{*},Q[-1]) \end{array}$$

and so


$$\begin{array}{r} \chi_{q^{1/2}}(H(M_{\left(1,1,1\right)}^{\zeta -ss}(Q),\phi_{\text{Tr}(W)}Q[3]))x^{\left(1,1,1\right)}=2\cdot \frac{-q^{1/2}}{1-q}. \end{array}$$

Now we consider the case of general $W=c_{1}b_{1}a_{1}+c_{2}b_{2}a_{2}+W_{\geq 6}$. In this case, we find that the scheme-theoretic critical locus of Tr(W) contains the points α and β as reduced connected components, since after a formal change of coordinates, we may transform $\text{Tr}(W)=c_{j}a_{i}+a_{i}b_{j}+(\text{higher order terms})$ back into the form (5). It follows that the restriction of $\phi_{\text{Tr}(W)}Q_{M}[4]$ to a small analytic neighbourhood of α is $Q_{\alpha }$, and its restriction to a small analytic neighbourhood of β is $Q_{\beta }$. Set N=M∖{α,β}. Passing to derived global sections, we find that there is a direct sum decomposition


$$H(M_{\left(1,1,1\right)}^{\zeta -ss}(Q),\phi_{\text{Tr}(W)}Q[4])≅H(\alpha \cup \beta ,Q)⊕H(N,\phi_{\text{Tr}(W)}Q[4]).$$

Then we set $e(q^{1/2})=\chi_{q^{1/2}}(H(N,\phi_{\text{Tr}(W)}Q[4]))$.∎

## 3. Counterexamples

### 3.1. Spherical (non) generation

We refer to [1, §2.4] for the definition of the shuffle algebra $H_{Q}$ associated to an arbitrary quiver. It is shown there that this shuffle algebra is isomorphic to the CoHA $H_{Q,W}$ with W=0. Let $d\in {\mathbb{N}}^{Q_{0}}$ be a dimension vector for Q. At the level of underlying vector spaces, we have


$$H_{Q,d}≅Q[z_{1,1},\ldots ,z_{1,d_{1}},z_{2,1},\ldots ,z_{Q_{0},1},\ldots ,z_{Q_{0},d_{Q_{0}}}{]}^{S_{d}},$$

where the symmetric group $S_{d}=\prod_{i\in Q_{0}}S_{d_{i}}$ acts by permuting all variables while preserving the first of their two subscripts. The cohomological degree of a homogeneous polynomial p(z) is given by setting


$$|p(z)|=2\deg(p(z))+\chi_{Q}(d,d).$$

We continue to denote by Q the Markov quiver from §2. We define the *spherical subalgebra* $S_{Q}\subset H_{Q}$ to be the subalgebra generated by all the subspaces $H_{Q,\delta_{i}}\subset H_{Q}$ for $i\in Q_{0}$. The algebra $S_{Q}$ inherits a ${\mathbb{N}}^{Q_{0}}$-grading from $H_{Q}$, and for $d\in {\mathbb{N}}^{Q_{0}}$, we define $S_{Q,d}\subset S_{Q}$ to be the dth graded piece. More generally, we define the spherical subalgebra $S_{Q,W}\subset H_{Q,W}$ to be the subalgebra generated by the subspaces $H_{Q,W,\delta_{i}}$ for $i\in Q_{0}$, and we denote by $S_{Q,W,d}\subset S_{Q,W}$ the dth graded piece. We say that $H_{Q,W}$ is spherically generated if it is equal to its spherical subalgebra.

The vector space $S_{Q,(1,1,1)}$ is spanned by shuffle products of polynomials $p_{1}(z_{1})$, $p_{2}(z_{2})$ and $p(z_{3})$, in any order, where we have abbreviated $z_{i}=z_{i,1}$ for i=1,2,3. By the formula for the shuffle product in [1, §2.4], we find


$$\begin{array}{rl} p_{3}(z_{3})\circ p_{2}(z_{2})\circ p_{1}(z_{1})= & \left(z_{1}-z_{3}{)}^{2}p_{1}(z_{1})p_{2}(z_{2})p_{3}(z_{3}\right)\\ p_{3}(z_{3})\circ p_{1}(z_{1})\circ p_{2}(z_{2})= & (z_{1}-z_{3}{)}^{2}(z_{2}-z_{1}{)}^{2}p_{1}(z_{1})p_{2}(z_{2})p_{3}(z_{3}), \end{array}$$

and the same formulas hold under cyclic permutation of the subscripts. We thus find that $S_{Q,(1,1,1)}$ is spanned as a vector space by elements


(3.1) $$(z_{1}-z_{3}{)}^{2}p,\;(z_{3}-z_{2}{)}^{2}r,\;(z_{2}-z_{1}{)}^{2}s,$$

where $p,r,s\in \mathbb{Z}[z_{1},z_{2},z_{3}]$. In particular, we find that


$$S_{Q,(1,1,1)}^{n}≅\left\{ \begin{array}{cc} 0 & \text{if }n<1\\ \mathbb{Q}\;\cdot \;(z_{1}-z_{3}{)}^{2}⊕︎\mathbb{Q}\;\cdot \;(z_{2}-z_{1}{)}^{2}⊕︎\mathbb{Q}\;\cdot \;(z_{3}-z_{2}{)}^{2} & \text{if }n=1. \end{array}\right.$$

Taking dimensions


$$\dim(S_{Q,(1,1,1)}^{n})=\left\{ \begin{array}{cc} 0 & \text{if }n<1\\ 3 & \text{if }n=1. \end{array}\right.$$

If instead we allow non-zero potential W, we find that we still have isomorphisms $H_{Q,W,\delta_{i}}≅Q[z_{i}]$, and via the cohomological degree calculation of (1.1), the following lemma:

**Lemma 3.1.** *For* Q *the Markov quiver and* W *arbitrary, we have*


$$S_{Q,W,(1,1,1)}^{n}=\left\{ \begin{array}{cc} 0 & \text{if }n<1\\ \mathrm{Span}(z_{1}^{0}\circ z_{3}^{0}\circ z_{2}^{0},\;z_{2}^{0}\circ z_{1}^{0}\circ z_{3}^{0},\;z_{3}^{0}\circ z_{2}^{0}\circ z_{1}^{0}) & \text{if }n=1. \end{array}\right.$$

Now we reinstate the assumption that W is generic. From the calculations of BPS invariants in §2.2, we calculate the dimensions of $H_{Q,W,d}^{n}$ for low values of d


$$\begin{array}{rl} Z_{Q,W}(x)= & \left(1+x^{\left(1,0,0\right)}\frac{-q^{1/2}}{1-q}\right)*\left(1+x^{\left(1,1,0\right)}(-q^{-1/2}-q^{1/2})\frac{-q^{1/2}}{1-q}\right)*\left(1+x^{\left(0,1,0\right)}\frac{-q^{1/2}}{1-q}\right)*\\ & *\left(1+(2+e(q^{1/2}))x^{\left(1,1,1\right)}\frac{-q^{1/2}}{1-q}\right)*\left(1+x^{\left(0,1,1\right)}(-q^{-1/2}-q^{1/2})\frac{-q^{1/2}}{1-q}\right)*\\ & *\left(1+x^{\left(0,0,1\right)}\frac{-q^{1/2}}{1-q}\right)+\text{higher order terms}, \end{array}$$

where the higher-order terms are linear combinations of monomials $x^{d}$ with at least one of $d_{1},d_{2},d_{3}>1$, and $e(q^{1/2})$ is the Laurent polynomial introduced in proposition 2.3. Write $u(q^{1/2})x^{\left(1,1,1\right)}$ for the $x^{\left(1,1,1\right)}$ term of $Z_{Q,W}(x)$. Then the above-mentioned factorization of $Z_{Q,W}(x)$ yields


$$\begin{array}{rl} u(q^{1/2})x^{\left(1,1,1\right)}=-q^{1/2} & (\frac{q}{(1-q{)}^{3}}x^{\left(1,0,0\right)}*x^{\left(0,1,0\right)}*x^{\left(0,0,1\right)}+\\ & +\frac{1+q}{(1-q{)}^{2}}(x^{\left(1,1,0\right)}*x^{\left(0,0,1\right)}+x^{\left(1,0,0\right)}*x^{\left(0,1,1\right)})+\\ & +\frac{2+e(q^{1/2})}{1-q}x^{\left(1,1,1\right)}) \end{array}$$

and so


(3.2) $$\begin{array}{lcr} & u(q^{1/2})=-q^{1/2}\frac{2-q^{2}+(2+e(q^{1/2}))(1-q{)}^{2}}{(1-q{)}^{3}}. & \end{array}$$

Observing that the coefficients of even powers of $q^{1/2}$ in $e(q^{1/2})$ are positive, we deduce that the modulus of the $q^{1/2}$ coefficient of $u(q^{1/2})$ is at least 4, and so, comparing with lemma 3.1, we deduce the following:

**Proposition 3.2.** *Let* Q *be the Markov quiver from §2. Let* $W=c_{1}b_{1}a_{1}+c_{2}b_{2}a_{2}+W_{\geq 6}$ *be a generic potential. Then* $H_{Q,W}$ *is not spherically generated. Moreover,* $\dim(H_{Q,W,(1,1,1)}^{1})>\dim(S_{Q,(1,1,1)}^{1})$.

In particular, there is no (graded) isomorphism $H_{Q,W}≅S_{Q}$.

### 3.2. Excluding non-spherical generators

Let G=ℤ/2⋅ℤ. Fix the potential $W=c_{1}b_{1}a_{1}+c_{2}b_{2}a_{2}$. We consider the G-action on Q that swaps $a_{1}$ with $a_{2}$, $b_{1}$ with $b_{2}$ and $c_{1}$ with $c_{2}$. This action fixes W. As such, G acts on the critical cohomology $H_{Q,W}$, and it is easy to see that the CoHA multiplication is G-equivariant. Furthermore, G acts trivially on $H_{Q,W,\delta_{i}}$ for i=1,2,3, and so G acts trivially on the *entire* spherical subalgebra. Therefore, letting $H_{Q,W}^{\mathrm{sgn}}\subset H_{Q,W}$ be the summand carrying the sign representation for G, elements of this summand are not spherically generated. The idea of isolating and excluding the BPS contributions that are not G-invariant has been explored already in the physics literature, for example, in [13, §4.4.5] and [14, §4.2].

With α and β the (1,1,1)-dimensional representations introduced in (2.2), the vector space $H(M_{\left(1,1,1\right)}^{\zeta -ss}(Q),\phi_{\text{Tr}(W)}Q_{M_{\left(1,1,1\right)}^{\zeta -ss}(Q)}[4])≅H(\alpha \cup \beta ,Q)$, which is the BPS cohomology giving rise to the BPS invariant $\Omega_{Q,W,(1,1,1)}^{\zeta }=2$, carries the regular G-representation. The Poincaré series $\Omega_{Q,W,(1,1,1)}^{\zeta ,G-inv}$ of the G-invariant part of the BPS cohomology is thus 1, and so repeating the calculation of (3.2), we find the generating function for the G-invariant part of the CoHA


$$\chi_{q^{1/2}}(H_{Q,W,(1,1,1)}^{G-inv})=-q^{1/2}\frac{2-q^{2}+(1-q{)}^{2}}{(1-q{)}^{3}}.$$

From (3.1), under the change of variables $x=z_{1}-z_{3},y=z_{3}-z_{2},z=z_{1}$*,* the ideal $S_{Q,(1,1,1)}\subset \mathbb{Q}[x,y,z]$ is identified with the ideal containing elements of degree at least 2 in x,y. So we have $S_{Q,(1,1,1)}≅\mathbb{Q}[x,y{]}_{\geq 2}⊗\mathbb{Q}[z]$*,* and


$$\begin{array}{rl} \chi_{q^{1/2}}(S_{Q,(1,1,1)})= & -q^{-3/2}((1-q{)}^{-2}-1-2q)(1-q{)}^{-1}\\ = & \chi_{q^{1/2}}(H_{Q,W,(1,1,1)}^{G-inv}). \end{array}$$

Put differently, the non-spherically generated part of $H_{Q,W,(1,1,1)}$ is given by elements $u^{n}\cdot (1_{\alpha }-1_{\beta })$, where $u\in \mathbb{Q}[u]=H(pt/{\mathbb{C}}^{*},\mathbb{Q})$ acts via multiplication by the first Chern class of the determinant line bundle. It is possible to show that the algebra generated by these elements surjects onto the free exterior algebra A generated by the same symbols. A physically motivated possible modification of the spherical generation conjecture, suggested by Davide Gaiotto, is that $H_{Q,W}$ splits as the product of A and $S_{Q}$. Via dimensional reduction [15, appendix A] and proposition 3.5, it should be possible to test this prediction for low-dimension vectors.

### 3.3. Dependence on W

The second part of proposition 3.2 provides a counterexample to the conjecture regarding spherical subalgebras in [4, §2.1]. A weaker conjecture, also stated in [4, §2.1], is that for infinitely mutable W, $H_{Q,W}$ is independent of W. Comparing the two parts of proposition 2.3, this would imply the equality $e(q^{1/2})=0$ for all W. To exclude the ‘error term’ $e(q^{1/2})$ one could instead conjecture that $H_{Q,W}^{\text{nilp}}$ is independent of W, where we define $\iota_{d}:M_{d}^{\text{nilp}}(Q)↪M_{d}(Q)$ to be the inclusion of the reduced substack containing the nilpotent representations, and


$$H_{Q,W,d}^{\text{nilp}}=H(M_{d}^{\text{nilp}}(Q),\iota_{d}^{!}\phi_{Tr(W)}Q[\chi_{Q}(d,d)]).$$

Defining $Z_{Q,W,\theta }^{\zeta ,\text{nilp}}(x)$ and $\Omega_{Q,W,d}^{\zeta ,\text{nilp}}$ starting from $H_{Q,W,d}^{\text{nilp}}$ as in (1.2) and (1.4), respectively, we may instead conjecture that the nilpotent BPS invariants $\Omega_{Q,W,d}^{\zeta ,\text{nilp}}$ are independent of W. The multiplication is again as defined in [1, §7]. Alternatively, one could conjecture that for quasi-homogeneous infinitely mutable potentials W, the CoHA $H_{Q,W}$ is independent of W. In this final section, on the one hand, we show that the Markov quiver provides counterexamples to these forms of the independence conjecture, but on the other hand, our results will indicate a way forward with a weakened version of the spherical generation conjecture.

We consider the ‘marginal’ potential $W_{marg}=c_{1}b_{1}a_{2}+c_{1}b_{2}a_{1}+c_{2}b_{1}a_{1}$—the homogeneous potential of type (4). Recall from proposition 2.2 that this potential is infinitely mutable.

**Lemma 3.3.** *There is an equality of generating series*


$$\chi_{q^{1/2}}(H_{Q,W_{marg},(1,1,1)})=-q^{1/2}\frac{3-2q}{(1-q{)}^{3}}.$$

*Proof*. By Verdier self-duality of the vanishing cycle sheaf, we have the isomorphism


$$H(M_{\left(1,1,1\right)}(Q),\phi_{\text{Tr}(W_{marg})}Q[-\chi_{Q}(d,d)])≅H_{c}(M_{\left(1,1,1\right)}(Q),\phi_{\text{Tr}(W_{marg})}Q[3]{)}^{\vee },$$

where the right-hand side is the graded vector dual of the compactly supported hypercohomology. Let $Q^{\prime }$ be the quiver obtained from Q by removing the arrows $c_{1}$ and $c_{2}$. Define


$$\begin{array}{rl} A:= & CQ^{\prime }/\langle \partial W_{marg}/\partial c_{1},\partial W_{marg}/\partial c_{2}\rangle \\ ≅ & CQ^{\prime }/\langle b_{1}a_{2}+b_{2}a_{1},a_{1}b_{1}\rangle . \end{array}$$

We denote by $M_{\left(1,1,1\right)}(A)$ the stack of (1,1,1)-dimensional A-modules. By the dimensional reduction isomorphism [[15], appendix A], there is an isomorphism


$$H_{c}(M_{\left(1,1,1\right)}(Q),\phi_{\text{Tr}(W_{marg})}Q)≅H_{c}(M_{\left(1,1,1\right)}(A),Q)[-2].$$

The stack $M_{\left(1,1,1\right)}(A)$ is isomorphic to the global quotient stack Z/T, where $Z\subset A^{4}$ is cut out by the equations $b_{1}a_{2}+b_{2}a_{1}=0$ and $a_{1}b_{1}=0$, $T=({\mathbb{C}}^{*}{)}^{3}$, and the first copy of ${\mathbb{C}}^{*}$ scales the $a_{i}$ coordinates, the second scales the $b_{i}$ coordinates and the third acts trivially. We define


$$\begin{array}{rl} U_{1}= & \left\{(a_{1},a_{2},b_{1},b_{2})\in Z\;|\;a_{1}=0,a_{2}=0\right\}\\ U_{2}= & \left\{(a_{1},a_{2},b_{1},b_{2})\in Z\;|\;a_{1}=0,a_{2}\neq 0\right\}\\ U_{3}= & \{(a_{1},a_{2},b_{1},b_{2})\in Z\;|\;a_{1}\neq 0\}. \end{array}$$

Then


$$\begin{array}{rl} U_{1}/T & ≅A^{2}/T\\ U_{2}/T & ≅A^{1}/({\mathbb{C}}^{*}{)}^{2}\\ U_{3}/T & ≅A^{1}/({\mathbb{C}}^{*}{)}^{2}. \end{array}$$

These three stacks stratify $M_{\left(1,1,1\right)}(A)$. All of the above-mentioned stacks have pure mixed Hodge structures on their compactly supported cohomology, so that their weight Poincaré series agree with their naive Poincaré series, and the above-mentioned stratification gives the identity


$$\begin{array}{rl} \chi_{q^{1/2}}(H_{c}(M_{\left(1,1,1\right)}(A),Q{)}^{\vee })= & \sum_{1\leq i\leq 3}\chi_{q^{1/2}}(H_{c}(U_{i}/T,Q{)}^{\vee })\\ = & {\left(\frac{q^{2}}{(q-1{)}^{3}}+\frac{2q}{(q-1{)}^{2}}\right)}_{q\mapsto q^{-1}} \end{array}$$

as required.∎

Comparing with the analogous calculation for the generic infinitely mutable potential $W_{gen}=c_{1}b_{1}a_{1}+c_{2}b_{2}a_{2}$ yields the following.

**Proposition 3.4.** *The CoHA* $H_{Q,W}$ *is not independent of the choice of infinitely mutable potential* W*. There are equalities of refined BPS invariants* $\Omega_{Q,W_{gen},(1,1,1)}^{\zeta }=2$ *and* $\Omega_{Q,W_{marg},(1,1,1)}^{\zeta }=1$*. Moreover, there is an equality* $\Omega_{Q,W_{marg},(1,1,1)}^{\zeta ,\text{nilp}}=1$.

*Proof*. Comparing lemma 3.3 with (3.2), we find that the $x^{\left(1,1,1\right)}$ coefficient of $Z_{Q,W_{gen}}(x)-Z_{Q,W_{marg}}(x)$ is given by


(3.3) $$\begin{array}{lcr} & -q^{1/2}\frac{\left(4-4q+q^{2})-(3-2q\right)}{(1-q{)}^{3}}=-q^{1/2}\frac{1}{1-q}. & \end{array}$$

Since this difference is non-zero, the graded dimensions of $H_{Q,W_{gen},(1,1,1)}$ and $H_{Q,W_{marg},(1,1,1)}$ are not the same.

The equality $\Omega_{Q,W_{gen},(1,1,1)}^{\zeta }=2$ is proposition 2.3. Since for dimension vectors $d^{\prime }$ strictly less than (1,1,1) in the natural partial order, we have $\Omega_{Q,W_{gen},d^{\prime }}^{\zeta }=\Omega_{Q,W_{marg},d^{\prime }}^{\zeta }$, it follows from (1.2) and (1.4) that (3.3) is equal to $-q^{1/2}\frac{\Omega_{Q,W_{gen},(1,1,1)}^{\zeta }-\Omega_{Q,W_{marg},(1,1,1)}^{\zeta }}{1-q}$.

Finally, we show that $\Omega_{Q,W_{marg},(1,1,1)}^{\zeta ,\text{nilp}}=\Omega_{Q,W_{marg},(1,1,1)}^{\zeta }$. We define $M_{i,j}$ for i,j∈{1,2} as in the proof of proposition 2.3. It is easily verified that for (i,j)≠(2,2), we have $\text{Crit}(\text{Tr}(W))\cap M_{i,j}=\emptyset$. On $M_{2,2}$, we have the affine coordinates $a_{1},b_{1},c_{1},c_{2}$, in which Tr(W) becomes $g=c_{1}b_{1}+c_{1}a_{1}+c_{2}a_{1}b_{1}$, which has critical locus (forgetting the scheme structure) given by $a_{1}=b_{1}=c_{1}=0$. Now we claim that there is an isomorphism


(3.4) $$H(M_{2,2},\phi_{g}Q[4])≅H(M_{2,2}^{\text{nilp}},i^{!}\phi_{g}Q[4]{)}^{\vee },$$

where $M_{2,2}^{\text{nilp}}$ is the locus cut out by the equations $c_{1}=c_{2}=0$ and $i:M_{2,2}^{\text{nilp}}↪M_{2,2}$ is the inclusion. Since on the right-hand side we have taken the graded dual, the claimed isomorphism implies that there is an equality


$$\Omega_{Q,W_{marg},(1,1,1)}^{\zeta ,\text{nilp}}=(\Omega_{Q,W_{marg},(1,1,1)}^{\zeta }{)}_{q^{1/2}\mapsto q^{-1/2}}=1_{q^{1/2}\mapsto q^{-1/2}}=1$$

as required. We have isomorphisms


$$\begin{array}{rl} H(M_{2,2}^{\text{nilp}},i^{!}\phi_{g}Q[4])≅ & H(M_{2,2}^{\text{nilp}},Di^{*}D\phi_{g}Q[4])\\ ≅ & H(M_{2,2}^{\text{nilp}},Di^{*}\phi_{g}Q[4])\\ ≅ & H(M_{2,2}^{\text{nilp}},i^{*}\phi_{g}Q[4]{)}^{\vee }, \end{array}$$

where D denotes the Verdier duality functor. The second isomorphism exists since the vanishing cycle sheaf is Verdier self-dual [16], while the third follows from the fact that $i^{*}\phi_{g}Q[4]$ has compact support.

Let ${\mathbb{R}}_{>0}$ act on $M_{2,2}^{\text{nilp}}$ by scaling $c_{1}$ with weight 1 and $c_{2}$ with weight 2. We extend this to an action on $M_{2,2}$ by letting ${\mathbb{R}}_{>0}$ act on $a_{1},b_{1}$ with weight −1. Then g is a ${\mathbb{R}}_{>0}$-invariant function, and $\phi_{g}Q[4]$ is a ${\mathbb{R}}_{>0}$-equivariant perverse sheaf. Let $\tau :A^{1}↪M_{2,2}$ be the inclusion of the critical locus of g, let $\iota :\{0\}↪A^{1}$ be the inclusion of the origin and let $G=\tau^{*}\phi_{g}Q[4]$. Since $\phi_{g}Q[4]$ is supported on the image of τ, we have isomorphisms


$$H(M_{2,2},\phi_{g}Q[4])≅H(A^{1},G);\;\;H(M_{2,2}^{\text{nilp}},i^{*}\phi_{g}Q[4])≅H(A^{1},\iota_{*}\iota^{*}G)$$

by base change. Since G is ${\mathbb{R}}_{>0}$-equivariant, it follows from [17], proposition 3.7.5] that the adjunction map $H(A^{1},G)\to H(A^{1},\iota_{*}\iota^{*}G)$ is an isomorphism. Putting all of these isomorphisms together, we obtain (3.4).∎

By proposition 3.4 and proposition 3.5 below, $H_{Q,W_{marg}}$ is spherically generated in degree (1,1,1). We note, following lemma 2.1, that this is a *non-generic* infinitely mutable potential. Whether spherical generation continues for the marginal potential, for higher-dimension vectors, is an interesting problem, which (via dimensional reduction) may again be tested numerically. More generally, an interesting modification of the conjecture in [4, §2.1] would be that *for every quiver, there exists at least one infinitely mutable potential for which the CoHA is spherically generated*.

We finish with a proposition that should be useful for studying this conjecture. Before stating it, we recall that a potential W is called *quasi-homogeneous* if there is a grading $p:Q_{1}\to \mathbb{N}$ such that all the cycles appearing in W are of the same total degree d with respect to the grading p and d>0.

**Proposition 3.5.** *Let* Q *be a quiver without loops, and let* W *be a quasi-homogeneous potential. Then* $H_{Q,W}≅S_{Q}$ *as graded algebras if and only if* $Z_{Q,W}(x)=\sum_{d\in N^{Q_{0}}}\chi_{q^{1/2}}(S_{Q,d})x^{d}$*. In this case,* $H_{Q,W}$ *is spherically generated*.

*Proof*. One implication is trivial: if two graded algebras are isomorphic, they certainly have the same graded dimensions. So we need to show the reverse implication. For this, we consider the morphism of CoHAs $\xi :H_{Q,W}\to H_{Q}$ constructed in [2, prop 4.4] (this uses that W is quasi-homogeneous). Since Q has no loops, it follows that Tr(W)=0 when restricted to each of the stacks $M_{\delta_{i}}(Q)$ for $i\in Q_{0}$. It follows that ξ induces an isomorphism when we restrict to the $\delta_{i}$ graded piece. In particular, the image of ξ contains $S_{Q}$. By the equality of graded dimensions, the image of ξ is precisely $S_{Q}$, and ξ induces an isomorphism $H_{Q,W}≅S_{Q}$. The final statement follows, since $S_{Q}$ is spherically generated by definition.∎

Via proposition 3.5, the kinds of calculations of BPS invariants performed in this paper may be used not just to test the variants of the spherical generation conjecture discussed above, but try to prove them.

## Data Availability

This article has no additional data.

## Footnotes

^1^
This is a kind of non-degeneracy condition arising in cluster algebras. Rather than spell out the definition, we refer to lemma 2.1 for examples and non-examples of infinitely mutable potentials.

^2^
Note that the new potential may be a formal linear combination of cyclic paths in Q.
